# Intracranial cystic chondroma: a case report

**DOI:** 10.1186/1752-1947-6-432

**Published:** 2012-12-28

**Authors:** Muhammad Muzaffer Uddin, Junaid Ashraf, Akhtar Amin Memon, Jamshed Ali

**Affiliations:** 1 Department of Neurosurgery, Civil Hospital Karachi, Karachi, Pakistan; 2 Dow Medical College, Dow University of Health Sciences, Karachi, Pakistan

**Keywords:** Cartilaginous tumor, Intracranial chondroma, Pakistan

## Abstract

**Introduction:**

Intracranial chondromas are rare benign tumors with an incidence of 0.2% to 0.3% of all intracranial tumors. This is the first case of an intracranial chondroma reported from Pakistan.

**Case presentation:**

We report a case of a 23-year-old Asian man presenting with intracerebral chondroma of the left frontal lobe, which was eroding the dura matter. The intracranial chondroma was completely removed by surgery.

**Conclusion:**

Intracranial chondromas are rare benign cartilaginous tumors. Through this case presentation we have discussed the diagnostic procedures, radiological and pathological findings. The purpose of presenting such a rare case is to develop awareness among clinicians and medical students and to highlight the requirement of immediate actions to ensure proper management of such cases.

## Introduction

Intracranial chondromas are exceedingly rare neoplasms, which grow slowly by expansion. They are cysts of chondroid tissue, and present at different regions within the cranial cavity especially the skull base [1]. Sellar [2], parasellar [3], intradural and especially falcine chondromas [4] have also been reported.

We present a case of an intracerebral cystic chondroma of the left frontal lobe in a 23-year-old man which was diagnosed by radiological findings and further confirmed through pathological reports. Frontal craniotomy was performed for total tumor resection. This is the first case of intracranial chondroma reported from Pakistan. We have discussed the clinical presentation, etiology, radiological features, histological features and treatment of the case with references to previously reported cases.

## Case presentation

A 23-year-old Asian man with a history of generalized tonic–clonic seizures for the past year was admitted to a government hospital in Karachi, Pakistan.

**Figure 1:**
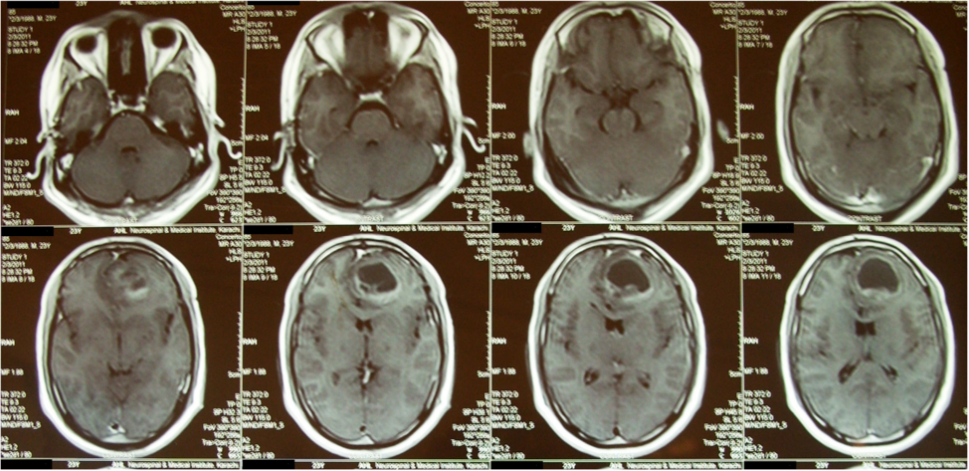
**Non-contrast T1-weighted axial magnetic resonance images revealing chondroma in the left frontal lobe.** Magnetic resonance images of the head conducted 3 days before surgery.

**Figure 2:**
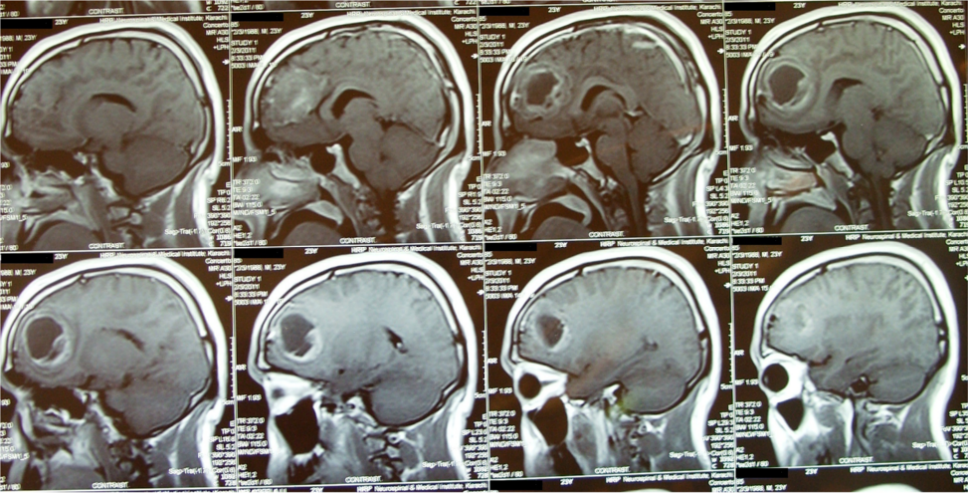
**Sagittal view of T-1 weighted magnetic resonance images with contrast revealing chondroma.** Sagittal magnetic resonance images of the brain conducted 3 days before surgery.

T1-weighted magnetic resonance imaging (MRI) revealed a space occupying lesion in the patient’s left frontal lobe (Figures 1, 2). T2-weighted MRI also reported similar findings.

A frontal craniotomy was performed. The tumor had eroded the dura mater. The tumor was hard and cystic, with the cavity containing greenish fluid. The whole of the cyst tumor was recovered. Hemostasis was secured. The dura was closed followed by closure of the pericranium.

Surgical pathology confirmed the case as intracranial cystic chondroma. The gross pathological report showed multiple tan, firm tissue fragments measuring 10.0×7.5×1.4cm. The surface appeared lobulated with focal areas of hemorrhage. The microscopic pathological report showed multiple fragments of chondroid tissue surrounded by a cyst wall. The outer surface of the cyst wall showed a thin fibrous capsule whereas the inner surface showed areas of hemorrhage. The chondroid tissue was composed of lobules of chondrocytes set in spaces against a chondroid matrix. Cells showed moderate cytoplasm, and the nuclei appeared round to oval and vesicular. A significant increase in cellularity, nuclear pleomorphism or mitosis was not noted. A few fragments of fibro-collagenous tissue with hemorrhage were also noted.

**Figure 3:**
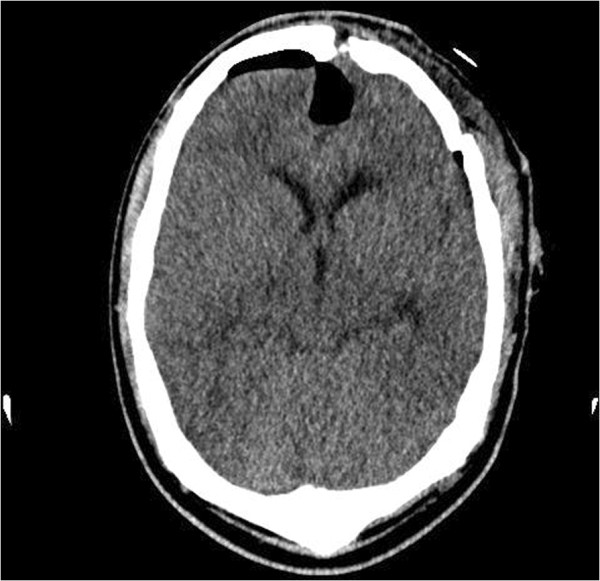
Post-operative computed tomography scan showing successful craniotomy.

Post-operative computed tomography (CT) scan showed successful resection of the tumor (Figure 3).

## Discussion

Intracranial chondromas are benign cartilaginous tumors. They are extremely rare and account for only 0.2% to 0.3% of all intracranial tumors [5,6]. Intracranial chondroma was first reported by Hirschfeld in 1851 [7]. These grow predominantly by expansion and do not invade the brain.

These tumors mostly originate from rests of cartilaginous cells at sphenoethmoidal and spheno-occipital synchondroses at the base of the skull [1]. They are also found in the sellar and parasellar regions, usually located extradurally [2,3]. In many cases, these tumors are intradural, developing mostly from the falx cerebri [4]. In extremely rare cases, these tumors might be intracerebral without any meningeal attachment [8]. In our case, the chondroma was intracerebral, originating in the left frontal lobe, but had expanded to erode the dura mater anteriorly and the falx cerebri medially.

Intracranial chondroma has also been reported as a component of Ollier’s multiple chondromatosis [9]. Pontine hemorrhage has also been associated with parasellar intracranial chondromas [3]. Association of skull base chondromas has also been reported with Maffucci syndrome [10].

Intracranial chondromas may develop in a person at any age but they have been most frequently observed in the third decade [7] which includes our case. Despite a purported lack of any sex predilection there are reports of a slight female predominance [11].

The presenting symptoms range from headaches [12] to lower cranial nerve palsy [13]. In some cases, proptosis, diplopia and varying degrees of visual activity impairment along with orbital extension have been reported [13]. Patients often complain of forgetfulness and lack of concentration [8].

Generalized tonic–clonic seizures are also usually the presenting complaints in intracranial chondromas [12], as in our case, which develop because of the gradual destruction of a large number of neurons that begin to fire at regular intervals. Focal neurological deficits may also result from mass effects of tumor [14].

Intracranial chondromas are thought to arise from mainly the skull base, dura, brain parenchyma or within the ventricles [12]. Various theories have been proposed to determine the etiology of intracranial chondromas but none has succeeded to ascertain a definite cause of origin. The most commonly accepted explanation for skull base chondromas is embryonic remnants of chondrogenic cells along the base [8]. The chondromas arising from the dura matter, choroid plexus, and cerebral cortex have been proposed to develop from metaplasia of meningeal fibroblasts and perivascular meninges [4]. Similarly, proliferation of ectopic embryologic rests of cartilage cells, traumatic displacement of cartilage elements or inflammatory cartilaginous activation of fibroblasts have been suggested to be the cause of development of intracranial chondromas [8].

Radiological findings of intradural chondromas are distinctive. On X-ray, intracranial chondromas represent hyperostosis of the internal table of the skull [7], enhanced intracranial pressure and calcified portions [15]. Intradural convexity chondromas possess carved, tufted, ring-shaped calcified areas [4].

A study reported that intradural chondromas possess two different CT appearances. The usually found type 1 shows mixed density with minimal or moderate enhancements. The rare type 2 shows an innermost less dense area containing a cyst [16]. Tanohata *et al*. reported two instances of skull base chondromas that exhibited delayed contrast enhancement on CT after a high-dose of the contrast medium was administered. They suggested this CT feature to be employed in differential diagnosis of intracranial chondromas from meningiomas and neurinomas [17].

MRI has become an important diagnostic tool for intracranial chondromas. Brownlee *et al*. reported variable signal intensity at different levels of MRI in a case of intracranial chondroma. At T1 they reported less intensity whereas at T2 the signal appeared to be of middle to high intensity [12].

The treatment of choice for such a benign space-occupying tumor is complete tumor excision, as in our case. The treatment recommended by Krayenbühl and Yasargil is total surgical removal of tumors because partial excision can improve the symptoms for a few years only [11]. Recurrence after total resection of the tumor is rarely reported. Moreover, there have been reports of recurrence after partial excision leading to malignant disintegration into chondrosarcoma. Thus, subtotal resection of the tumor may be mandatorily seconded with long-term radiological follow up.

## Conclusion

Concluding the above discussion, intracranial chondromas are masses of well-differentiated, cytologically benign cartilage having a small tendency for sarcomatous change and they do not invade the surrounding parenchyma. Treatment of choice is complete tumor resection with a favorable long-term prognosis.

## Consent

Written informed consent was obtained from the patient for publication of this case report and accompanying images. A copy of the written consent is available for review by the Editor-in-Chief of this journal.

## Competing interests

The authors declare that they have no competing interests.

## Authors’ contributions

The patient was under the care of MMU. MMU and JA surgically operated on the patient. MMU and AAM analyzed and interpreted the data. AAM wrote the manuscript. JA made additions to the manuscript. All authors reviewed and approved the final manuscript.
